# The prognosis of breast cancer patients with bone metastasis could be potentially estimated based on blood routine test and biochemical examination at admission

**DOI:** 10.1080/07853890.2023.2231342

**Published:** 2023-07-03

**Authors:** Bo Huang, Fang-Cai Wu, Wei-Dong Wang, Bu-Qing Shao, Xiao-Mei Wang, Ying-Miao Lin, Guo-Xing Zheng, Ming-Ming Dong, Can-Tong Liu, Yi-Wei Xu, Xin-Jia Wang

**Affiliations:** a Department of Orthopedics, The Cancer Hospital of Shantou University Medical College, Shantou, China; b Department of Orthopedics, The Second Affiliated Hospital of Shantou University Medical College, Shantou, China; c Department of Radiation Oncology, The Cancer Hospital of Shantou University Medical College, Shantou, China; d Esophageal Cancer Prevention and Control Research Center, the Cancer Hospital of Shantou University Medical College, Shantou, China; e Department of Clinical Laboratory Medicine, The Cancer Hospital of Shantou University Medical College, Shantou, China

**Keywords:** Breast cancer, bone metastasis, prognosis, nomogram, blood routine and biochemical examination

## Abstract

**Purpose:**

Due to the poor and unpredictable prognosis of breast cancer (BC) patients with bone metastasis, it is necessary to find convenient and available prognostic predictors. This study aimed to recognize the clinical and prognostic factors related to clinical laboratory examination and to construct a prognostic nomogram for BC bone metastasis.

**Methods:**

We retrospectively analyzed 32 candidate indicators from clinical features and laboratory examination data of 276 BC patients with bone metastasis. Univariate and multivariate regression analyses were performed to identify significant prognostic factors related to BC with bone metastasis. Nomogram was constructed and estimated by receiver operating characteristic (ROC) curves, calibration curves, and decision curve analysis.

**Results:**

Patients were randomly grouped into training (*n* = 197) and validation cohorts (*n* = 79). In training cohort, the multivariate regression analysis revealed that age, other organ metastasis sites, serum level of lactate dehydrogenase, globulin, white blood cell count, mean corpuscular volume, mean corpuscular hemoglobin, and monocyte ratio were independent prognostic factors for BC with bone metastasis. The prognostic nomogram in training cohort exhibited areas under the ROC curve (AUCs) of 0.797, 0.782, and 0.794, respectively, for predicting 1-, 3-, and 5-year overall survival. In validation cohort, the nomogram still showed acceptable discrimination ability (AUCs: 0.723, 0.742, and 0.704) and calibration.

**Conclusion:**

This study constructed a novel prognostic nomogram for BC patients with bone metastasis. It could serve as a potential tool of survival assessment to help individual treatment decision-making for clinicians.

## KEY MESSAGES

1. Our study investigated potential prognostic value of indicators from biochemical and blood routine examination for breast cancer patients with bone metastasis.
2. Our study established a nomogram based on the indicators from biochemical and blood routine examination, which might enhance the ability to predict prognosis of breast cancer patients with bone metastasis.

## Introduction

Breast cancer (BC), the most frequently diagnosed malignancy, is one of the main causes of cancer death in women [1]. Metastasis occurs in approximately 50% of BC patients. Bone is the most common metastasis site, a major cause of morbidity and impaired quality of life in BC patients. Significantly, bone metastasis account for 60%–70% in advanced BC patients [2]. Many complications including severe pain, pathological fractures, hypercalcemia, and spinal cord compression can be observed in BC patients with bone metastases [3]. The life quality for BC patients with bone metastasis can be slightly improved by various treatment modalities, such as chemotherapy, hormone therapy, and targeted therapy, since bone metastasis is an incurable status [4,5]. Therefore, predicting prognosis is in urgent need for personalized therapy strategy to BC patient with bone metastasis.

Currently, the prognostic predictors of bone metastasis include the features of original tumor, tumor markers, expression of selected genes, and related clinical manifestations [6–8]. Are there any more convenient examination indicators that can make clinicians to preliminarily judge the prognosis of BC bone metastasis for patients at the early stage of hospitalization? Nowadays, in order to predict the outcome and guide the clinical management, more and more researchers prefer to establish clinical prognosis models [9,10]. As a popular quantitative predictive tool, nomogram was usually used to predict the prognosis of cancer patients [7,11,12]. Recently, Mao et al. [13] have constructed a nomogram in combination with peripheral blood signatures and selected clinical characteristics, which predicted the prognosis of individual patients with nasopharyngeal carcinoma.

Increasing studies demonstrated that indicators from blood routine test and biochemical examination might be used for prognostic prediction of cancers [14–16]. For almost all cancer patients, blood routine and biochemical examination are convenient, quick, and inexpensive examinations, from which the information about human metabolism, inflammation, and internal environmental conditions can be obtained. There are limited studies on the application of blood routine and biochemical indicators in prognosis prediction of BC patients with bone metastasis. Thus, the purpose of our study was to identify the clinical and prognostic factors related to laboratory examination and to construct a prognostic nomogram for BC patients with bone metastasis, which were available for clinicians to initially predict the prognosis.

## Materials and methods

### Research design and patient population

**Figure 1.**
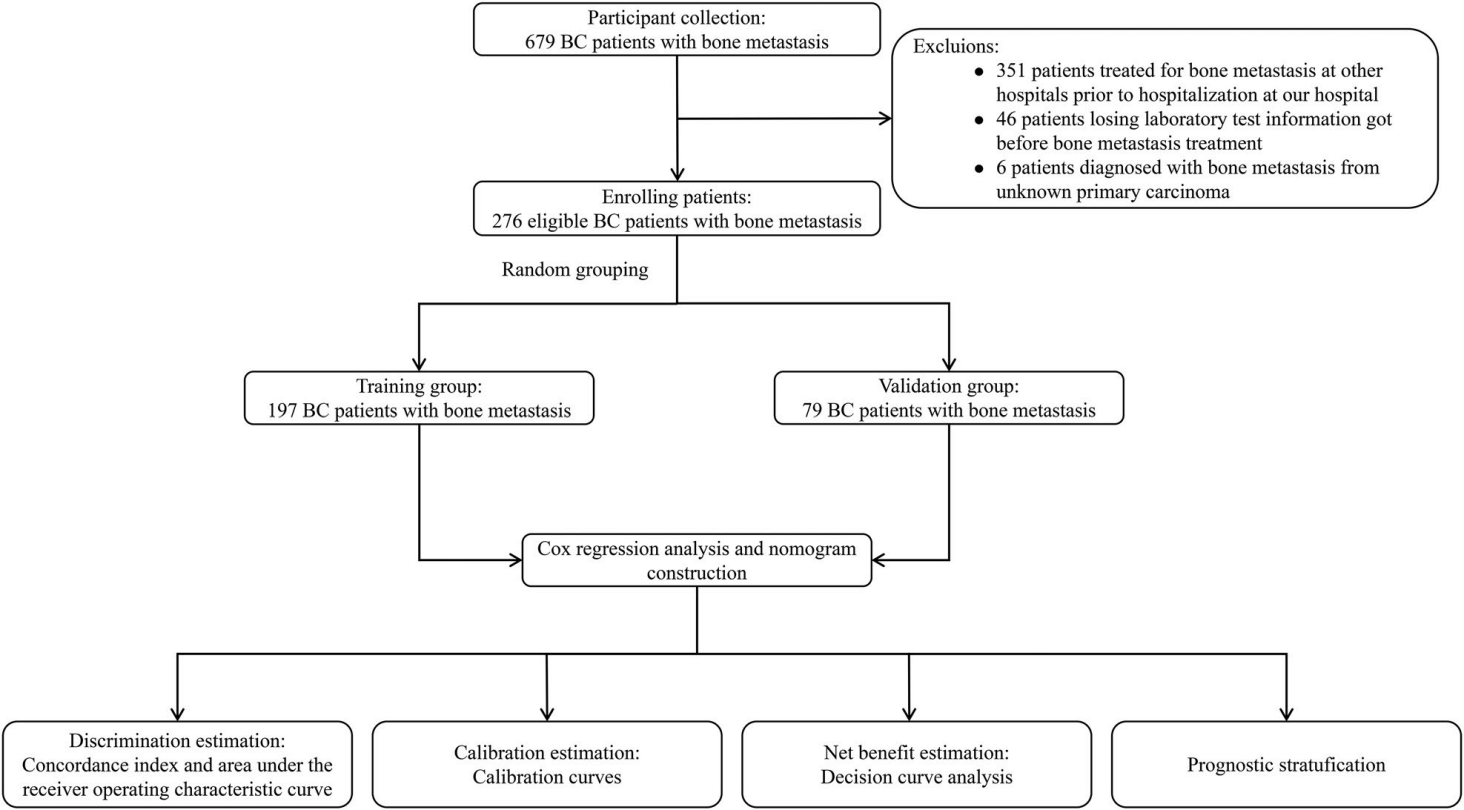
Flow chart of the study.

This was a retrospective study based on patients’ records. From 2010 to 2022, 679 BC patients with bone metastasis were treated in the Cancer Hospital of Shantou University Medical College. We applied strict inclusion and exclusion criteria during the enrollment process. Patients met the following inclusion criteria: (1) were diagnosed as primary breast cancer with histopathological analysis; (2) had no other primary tumors; (3) were confirmed with bone metastasis by bone scan and/or CT [8,17]. Moreover, patients with other organ metastases were diagnosed by plain radiographs, CT, and/or MRI. Patients who had previously received treatment for bone metastasis at other hospitals prior to hospitalization at our institution were excluded. Additionally, we excluded patients who were missing some laboratory test information before bone metastasis treatments and those who were diagnosed with bone metastasis from an unknown primary carcinoma. Finally, the remaining 276 patients met all the eligibility criteria for our study (Figure 1).

In the enrolled patients, comprehensive datasets for the relevant parameters (including clinical factors, results of laboratory examination before treatment of bone metastasis, and survival information) were provided. The clinical factors that may be relevant to prognosis were collected, including age, treatment of primary tumor, estrogen receptor (ER) status, progesterone receptor (PR) status, human epidermal growth factor receptor 2 (Her2) status, histological type, T stage, lymph node metastasis, liver metastasis, brain metastasis and lung metastasis. The laboratory examination indicators contained blood routine and biochemical examination. Blood routine included white blood cell count (WBC), red blood cell count (RBC), hemoglobin (HGB), red blood cell specific volume (HCT), mean corpuscular volume (MCV), mean corpuscular hemoglobin (MCH), mean corpuscular hemoglobin concentration (MCHC), platelet count (PLT), lymphocyte ratio (LY%), monocyte ratio (MO%), and neutrophil ratio (NE%). Biochemical examination enrolled lactate dehydrogenase (LDH), alkaline phosphatase (ALP), γ-glutamyl transpeptidase (GGT), total protein (TP), albumin (ALB), globulin (GLB), glucose (GLU), blood urea nitrogen (BUN), creatinine (CREAT), uric acid (UA), calcium (Ca), and serum phosphonium (Pi). In this study, the primary endpoint was overall survival (OS), calculated from the date of BC bone metastasis diagnosis to death or last follow-up time.

### Variable selection and nomogram construction

We randomly partitioned the dataset into training and validation cohorts using R programming language. According to clinical significance, we screened a total of 33 prognostic-related variables in the first step, which consisted of 10 categorical variables and 23 continuous variables. The optimal cut-off values for these continuous variables were then determined using X-tile software (Version 3.6.1, Yale University), following which they were converted into categorical variables. The cut-off values obtained from the X-tile analysis were utilized to stratify patients into high-risk and low-risk groups based on the values of age, LDH, ALP, GGT, TP, ALB, GLB, GLU, BUN, CREAT, UA, CA, Pi, WBC, RBC, HGB, HCT, MCV, MCH, MCHC, PLT, LY%, MO%, and NE%. The corresponding cut-off values were as follows: 58 years, 357 U/L, 349 U/L, 125 U/L, 79.5 g/L, 37.09 g/L, 28.09 g/L, 5.09 mmol/L, 5.92 mmol/L, 67.99 μmol/L, 357 μmol/L, 2.34 mmol/L, 1.31 mmol/L, 10.3×10^9^/L, 4.65×10^12^/L, 99.99 g/L, 0.34, 78.55 fl, 25.24 pg, 320.99 g/L, 272×10^9^/L, 16.42%, 11.42%, and 69.79%.

Cox proportional hazards regression analysis was performed using SPSS software to calculate the hazard ratio (HR) and 95% confidence interval (CI) for factors associated with OS. First, the variables with *p* values less than 0.05 were screened in the univariate Cox regression analysis, and then multivariate Cox proportional hazards regression analysis was performed based on those variables to construct a prognostic predictive model by a likelihood ratio backward stepwise regression method with a step probability ranging from 0.05 to 0.1.

Based on the Cox regression model, we constructed a nomogram for the prediction of 1-, 3- and 5-year OS rates in BC patients with bone metastases by the package of *rms* in R (https://CRAN.R-project.org/package=rms).

### Nomogram evaluation

To evaluate the discriminative ability, we constructed a receiver operating characteristic (ROC) curve. The nomogram discrimination efficacy was assessed by concordance index (C-index) and the area under the ROC curve (AUC). They basically ranged from 0.5 (random prediction) to 1.0 (excellent prediction). In general, a value greater than 0.7 indicated that the performance of model was good with moderate prediction capacity [18]. A ROC curve also contained the information about accuracy, sensitivity, and specificity [19]. Calibration curves were constructed by using a bootstrap method for 1000 resamples to compare the compatibility of the nomogram-predicted OS and the actual OS. Meanwhile, we depicted a decision curve analysis (DCA) to show the net benefit of different models [20].

Finally, we used the β-coefficients derived from the final Cox model to weight individual factors and then summed all of their contribution to generate raw prognostic scores. To normalize these subsequent scores to a range between 0 and 5, we employed the following equation: (5–0) × (raw prognostic score – min_score)/(max_score – min_score) + 0 [21]. In this equation, ‘min_score’ and ‘max_score’ represent the minimum and maximum values of the raw prognostic score, respectively. Then, we used median value of the normalized prognostic scores as cut-off value to divide the entire dataset into two prognostic risk groups and generated Kaplan-Meier survival curves and log-rank test.

### Statistical analysis

In this study, all analyses, figures and tables were performed by IBM SPSS Statistics 26 (La Jolla, CA, USA), Microsoft Excel (Redmond, WA, USA), X-tile version 3.6.1 (Version 3.6.1, Yale University) and R version 4.1.3 (https://www.r-project.org/). Descriptive statistics were used to summarize qualitative data as the number of cases (percentage), while quantitative data were reported as mean ± standard deviation or median (interquartile range), depending on their distribution normality. The intergroup comparisons of qualitative data were assessed by the chi-square test or Fisher’s exact test. For quantitative data that conformed to a normal distribution, two-sample independent Student’s t-test was employed, whereas those with a skewed distribution were compared with the Mann-Whitney U test. ROC curves and AUC were generated by the *timeROC* package in R (https://CRAN.R-project.org/package=timeROC). DCA was performed by the *ggDCA* package in R (https://CRAN.R-project.org/package=ggDCA). Kaplan-Meier survival curves were plotted by the *survival* and *survminer* packages in R (https://CRAN.R-project.org/package=survival). A *p* value of < 0.05 (two-tailed) was considered statistically significant for all analyses.

## Results

### Baseline characteristics

After patients’ enrollment, we analyzed the demographic and clinical characteristic of 276 enrolled patients (Table 1). The median follow-up time of the entire patients was 59 months (inter-quartile range (IQR): 34–94 months). The median age at the diagnosis of BC bone metastasis was 53 years old (IQR: 45–60). In total, 64.5% (178 patients) had multiple metastasis, and 44.2% (122 patients) had more than two kinds of organ metastasis. Meanwhile, 52.9% (146 patients) had underwent surgery in primary tumors, 68.1% (188 patients) had positive ER status, and 39.5% (109 patients) were in T3-4 stage. In the dataset, histology type of patients was mainly characterized as invasive ductal carcinoma (IDC), accounting for 92.4% (255 patients). In this study, the entire patients were randomly split at a ratio of 7:3 into the training (197 patients) and validation cohorts (79 patients).

**Table 1.** Patient characteristics in the study.

Categorical variables	Entire cohort (*n* = 276)	Training cohort (*n* = 197)	Validation cohort (*n* = 79)	Continuous variables	Entire cohort (*n* = 276)	Training cohort (*n* = 197)	Validation cohort (*n* = 79)
	Values (%)	Values (%)	Values (%)		Median (IQR)/Mean ± SD	Median (IQR)/Mean ± SD	Median (IQR)/Mean ± SD
Treatment for breast cancer				Laboratory test indicators			
Radiotherapy	4 (1.4)	3 (1.5)	1 (1.3)	LDH (U/L)	225.6 (183–348.3)	225 (183–327)	241 (185.05–523)
Chemotherapy	233 (84.4)	168 (85.2)	65 (82.3)	ALP (U/L)	124.5 (90.0–199.25)	118.9 (88.8–192)	133 (97–223.5)
Endocrine therapy	9 (3.3)	7 (3.5)	2 (2.5)	GGT (U/L)	32.05 (19–74.5)	31 (19–64)	38 (17.7–152)
Surgery	146 (52.9)	104 (52.8)	42 (53.1)	TP (g/L)	71.53 ± 7.41	71.92 ± 7.63	71.53 ± 6.82
More than 2 treatments	10 (3.6)	7 (3.5)	3 (3.8)	ALB (g/L)	41.0 (37.6–44.4)	41 (38.2–44.4)	40.9 (37.05–44.5)
No treatment	9 (3.3)	3 (1.5)	6 (7.6)	GLB (g/L)	31.17 ± 5.98	31.25 ± 6.19	31.17 ± 5.44
Histological type				GLU (mmol/L)	5.5 (4.97–6.25)	5.55 (5.01–6.22)	5.42 (4.96–6.34)
Invasive ductal carcinoma	255 (92.4)	177 (89.8)	78 (98.7)	BUN (mmol/L)	4.32 (3.42–5.60)	4.17 (3.33–5.48)	4.45 (3.54–5.89)
Others	16 (5.8)	15 (7.6)	1 (1.3)	CREAT (μmol/L)	73.42 ± 23.11	72.89 ± 21.96	74.76 ± 25.69
Unknown	5 (1.8)	5 (2.5)	–	UA (μmol/L)	358.56 ± 131.26	354.26 ± 118.91	358.56 ± 157.87
Other organ metastasis				CA (mmol/L)	2.3 (2.2–2.4)	2.3 (2.19–2.39)	2.3 (2.23–2.46)
Lung metastasis	21 (7.6)	15 (7.6)	6 (7.6)	Pi (mmol/L)	1.15 ± 0.25	1.13 ± 0.24	1.15 ± 0.26
Liver metastasis	31 (11.2)	21 (10.7)	10 (12.7)	WBC (×10^9^/L)	7.34 ± 4.93	7.29 ± 5.33	7.46 ± 3.75
Brain metastasis	4 (1.4)	3 (1.5)	1 (1.3)	RBC (×10^12^/L)	4.2 (3.7–4.62)	4.21 (3.7–4.65)	4.12 (3.73–4.46)
More than 2 kinds of organ metastasis	122 (44.2)	86 (43.7)	36 (45.6)	HGB (g/L)	119.8 (108.88–129.65)	119.7 (111.7–130.7)	120.5 (105–129.05)
Only-bone metastasis	98 (35.5)	72 (36.5)	26 (32.9)	HCT	0.37 (0.34–0.41)	0.38 (0.34–0.40)	0.37 (0.34–0.42)
Molecular subtype				MCV (fl)	88.01 ± 7.81	87.6 ± 7.21	88.01 ± 9.11
Her2+	135 (48.9)	99 (50.2)	36 (45.5)	MCH (pg)	29 ± 3.32	28.48 ± 3.49	29 ± 2.82
ER+	188 (68.1)	134 (68.0)	54 (68.3)	MCHC (g/L)	327.08 ± 12.6	327.05 ± 12.68	327.18 ± 12.39
PR+	163 (59.0)	110 (55.8)	53 (67.0)	PLT (×10^9^/L)	256.11 ± 100.6	261.2 ± 99.3	256.11 ± 103.67
T stage				LY%	26.28 ± 11.33	24.82 ± 9.96	26.28 ± 14.14
T1-2	140 (50.7)	98 (49.7)	42 (53.1)	MO%	8.03 ± 3.77	8.08 ± 3.8	8.03 ± 3.69
T3-4	109 (39.5)	78 (39.6)	31 (39.2)	NE%	65.3 ± 11.52	65.03 ± 11.83	65.3 ± 10.7
Unknown	27 (9.8)	21 (10.7)	6 (7.6)	age (years)	52.35 ± 10.80	53.05 ± 10.51	50.6 ± 11.32
Lymph node metastasis							
No	48 (17.4)	31 (15.7)	17 (21.5)				
Yes	219 (79.3)	159 (80.7)	60 (75.9)				
Unknown	9 (3.2)	7 (3.6)	2 (2.5)				

IQR: interquartile range**;** SD: standard deviation; ER: estrogen receptor; PR: progesterone receptor; Her2: human epidermal growth factor receptor 2; LDH: lactate dehydrogenase; ALP: alkaline phosphatase; GGT: γ-glutamyl transpeptidase; TP: total protein; ALB: albumin; GLB: globulin; GLU: glucose; BUN: blood urea Nitrogen; CREAT: creatinine; UA: uric acid; Ca: calcium; Pi: serum phosphonium; WBC: white blood cell count; RBC: red blood cell count; HGB: hemoglobin; HCT: red blood cell specific volume; MCV: mean corpuscular volume; MCH: mean corpuscular hemoglobin; MCHC: mean corpuscular hemoglobin concentration; PLT: platelet; LY%: lymphocyte ratio; MO%: Monocyte ratio; NE%: neutrophil ratio; Surgery: surgery of the primary site; Radiation therapy: radiation of the primary site.

### Cox proportional hazards regression analysis

**Figure 2.**
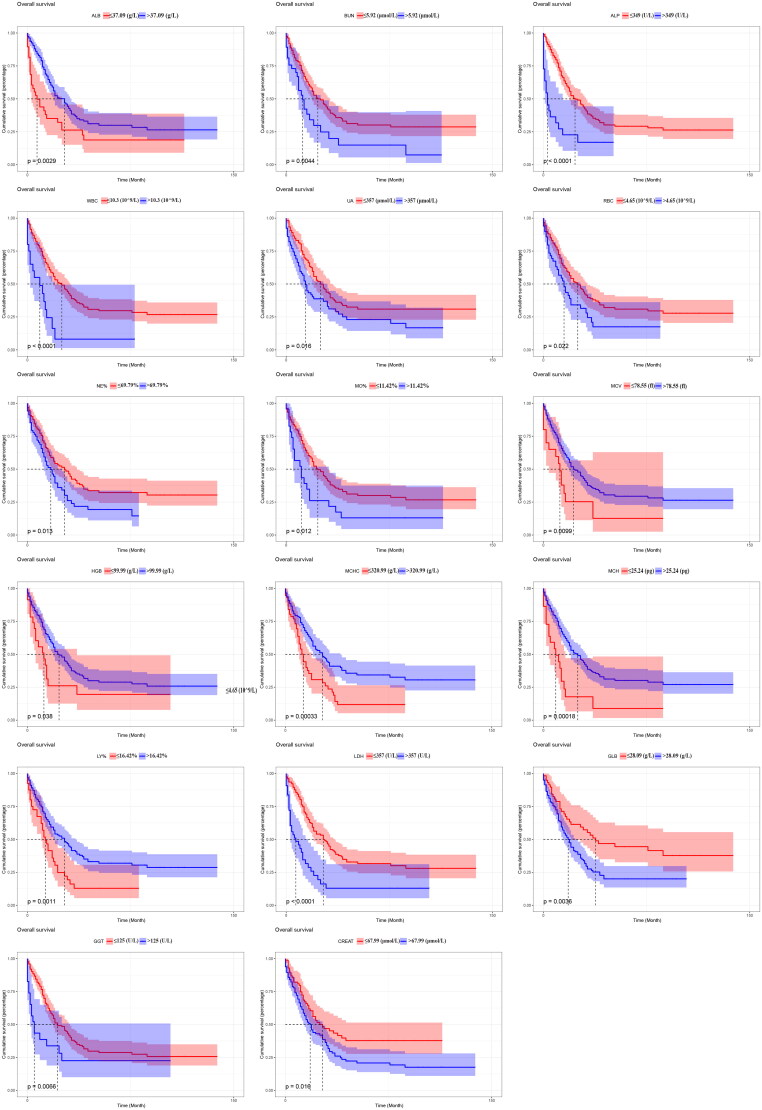
Kaplan-Meier survival curve to show that the different levels of laboratory examination indicators had a significant impact on the OS of patients.

In the training cohort, after univariate Cox regression analysis, 20 variables were significantly associated with OS, including age, ER status, other organ metastasis, and some laboratory test factors consisting of LDH, ALP, GGT, ALB, GLB, BUN, CREAT, UA, WBC, RBC, HGB, MCV, MCH, MCHC, LY%, MO%, and NE%. We drafted Kaplan-Meier survival curve to show that the different levels of laboratory examination indicators had significant impacts on the OS of patients (all *p* < 0.05, Figure 2).

After controlling for confounding variables with multivariate Cox regression, a prognostic model was constructed. In this model (Table 2), there are 8 variables were identified as independent prognostic factors, including age (HR:1.975; 95% CI: 1.350–2.888; *p* < .001), other organ metastasis (HR: 1.532; 95% CI: 1.015–2.313; *p* = .042), LDH (HR: 2.292; 95% CI: 1.467–3.581; *p* < .001), GLB (HR: 1.927; 95% CI: 1.245–2.982; *p* = .003), WBC (HR: 2.975; 95% CI: 1.641–5.395; *p* < .001), MCV (HR: 4.035; 95% CI: 1.136–14.332; *p* = .031), MCH (HR: 0.180; 95% CI: 0.054–0.592; *p* = .005), and MO% (HR: 2.675; 95% CI: 1.504–4.758; *p* = .001).

**Table 2.** Univariate and multivariate Cox regression analysis in breast cancer patients with bone metastases.

Variables	Univariate analysis		Multivariate analysis	
	HR (95%CI)	*p* value	HR (95%CI)	*p* value
Surgery				
No	Ref.			
Yes	1.114 (0.786–1.580)	.545		
Chemotherapy				
No	Ref			
Yes	1.115 (0.660–1.883)	.684		
Histological type				
No	Ref.			
Yes	1.143 (0.598–2.187)	.686		
Other organ metastasis				
No	Ref.		Ref.	
Yes	1.978 (1.340–2.919)	**.001**	1.532 (1.015–2..313)	**.042**
Age				
≤58	Ref.		Ref.	
>58	1.620 (1.132–2.317)	**.008**	1.975 (1.350–2.888)	**<.001**
T stage				
T1-2	Ref.			
T3-4	0.872 (0.601–1.265)	.469		
Lymph node metastasis				
No	Ref.			
Yes	1.171 (0.717–1,913)	.529		
Her2 status				
Negative	Ref.			
Positive	1.001 (0.706–1.417)	.997		
ER status				
Negative	Ref.		Ref.	
Positive	0.644 (0.448–0.925)	**.017**	0.712 (0.477–1.063)	.096
PR status				
Negative	Ref.			
Positive	0.828 (0.585–1.171)	.286		
LDH				
≤357 (U/L)	Ref.		Ref.	
>357 (U/L)	2.574 (1.728–3.833)	**<.001**	2.292 (1.467–3.581)	**<.001**
ALP				
≤349 (U/L)	Ref.		Ref.	
>349 (U/L)	2.703 (1.636–4.465)	**<.001**	1.296 (0.736–2.281)	.369
GGT				
≤125 (U/L)	Ref.		Ref.	
>125 (U/L)	1.987 (1.190–3.316)	**.009**	0.956 (0.408–2.242)	.918
TP				
≤79.5 (g/L)	Ref.			
>79.5 (g/L)	0.609 (0.327–1.131)	.116		
ALB				
≤37.09 (g/L)	Ref.		Ref.	
>37.09 (g/L)	0.541 (0.357–0.820)	**.004**	0.990 (0.955–1.026)	.574
GLB				
≤28.09 (g/L)	Ref.		Ref.	
>28.09 (g/L)	1.808 (1.200–2.725)	**.005**	1.927 (1.245–2.982)	**.003**
GLU				
≤5.09 (mmol/L)	Ref.			
>5.09 (mmol/L)	1.426 (0.969–2.099)	.072		
BUN				
≤5.92 (μmol/L)	Ref.		Ref.	
>5.92 (μmol/L)	1.848 (1.196–2.856)	**.006**	1.417 (0.873–2.301)	.159
CREAT				
≤67.99 (μmol/L)	Ref.		Ref.	
>67.99 (μmol/L)	1.547 (1.078–2.221)	**.018**	1.155 (0.774–1.724)	.481
UA				
≤357 (μmol/L)	Ref.		Ref.	
>357 (μmol/L)	1.526 (1.074–2.167)	**.018**	1.406 (0.952–2.007)	.087
CA				
≤2.34 (mmol/L)	Ref			
>2.34 (mmol/L)	1.365 (0.948–1.963)	.094		
Pi				
≤1.31 (mmol/L)	Ref.			
>1.31 (mmol/L)	1.361 (0.904–2.050)	.14		
WBC				
≤10.3 (109/L)	Ref.		Ref.	
>10.3 (109/L)	2.799 (1.641–4.773)	**<.001**	2.975 (1.641–5.395)	**<.001**
RBC				
≤4.65 (109/L)	Ref.		Ref.	
>4.65 (109/L)	1.560 (1.058–2.300)	**.025**	1.244 (0.805–1.922)	.325
HGB				
≤99.99 (g/L)	Ref.		Ref.	
>99.99 (g/L)	0.590 (0.353–0.985)	**.044**	0.869 (0.461–1.638)	.664
HCT				
≤0.34 %	Ref.			
>0.34 %	0.772 (0.523–1.139)	.192		
MCV				
≤78.55 (fl)	Ref.		Ref.	
>78.55 (fl)	0.501 (0.291–0.862)	**.013**	4.035 (1.136–14.332)	**.031**
MCH				
≤25.24 (pg)	Ref.		Ref.	
>25.24 (pg)	0.398 (0.240–0.660)	**<.001**	0.180 (0.054–0.592)	**.005**
MCHC				
≤320.99 (g/L)	Ref.		Ref.	
>320.99 (g/L)	0.519 (0.359–0.751)	**<.001**	0.680 (0.440–1.050)	.082
PLT				
≤272 (109/L)	Ref.			
>272 (109/L)	0.818 (0.566–1.184)	.287		
LY**%**				
≤16.42%	Ref.		Ref.	
>16.42%	0.525 (0.352–0.783)	**.002**	1.028 (0.963–0.598)	.921
MO**%**				
≤11.42%	Ref.		Ref.	
>11.42%	1.811 (1.122–2.921)	**.015**	2.675 (1.504–4.758)	**.001**
NE%				
≤69.79%	Ref.		Ref.	
>69.79%	1.558 (1.089–2.228)	**.015**	1.479 (0.954–2.294)	.080

HR: hazard ratio; CI: confidence interval; ER: estrogen receptor; PR: progesterone receptor; Her2: human epidermal growth factor receptor 2; LDH: lactate dehydrogenase; ALP: alkaline phosphatase; GGT: γ-glutamyl transpeptidase; TP: total protein; ALB: albumin; GLB: globulin; GLU: glucose; BUN: blood urea Nitrogen; CREAT: creatinine; UA: uric acid; Ca: calcium; Pi: serum phosphonium; WBC: white blood cell count; RBC: red blood cell count; HGB: hemoglobin; HCT: red blood cell specific volume; MCV: mean corpuscular volume; MCH: mean corpuscular hemoglobin; MCHC: mean corpuscular hemoglobin concentration; PLT: platelet; LY%: lymphocyte ratio; MO%: Monocyte ratio; NE%: neutrophil ratio; Surgery: surgery of the primary site; Radiation therapy: radiation of the primary site. The bold values were considered statistically significant.

### Construction of 1-, 3-, and 5-year OS predicting nomogram

**Figure 3.**
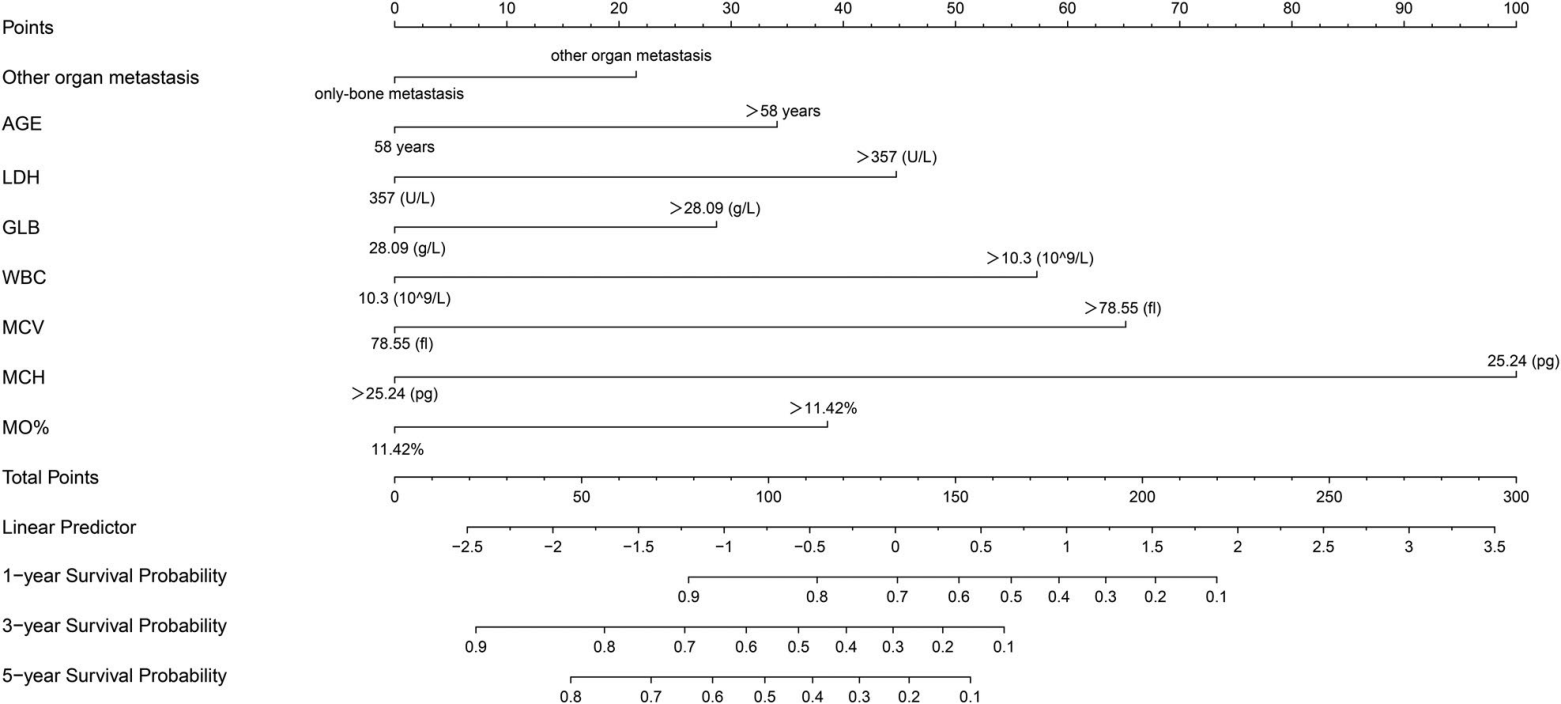
Nomogram for 1, 3, and 5-year OS prediction of the breast cancer patients with bone metastasis. Each prognostic factor was assigned a point on the scale, and the sum of the total points projected on the bottom scale represent the probabilities of 1, 3, and 5-year OS.

Based on the prognostic factors selected in the training cohort, a nomogram was developed for the prediction of OS (Figure 3). This prognostic nomogram is a predictive tool for estimating the 1-, 3-, or 5-year overall survival of patients with breast cancer bone metastasis. Each variable in the nomogram is assigned a point scale that represents its relative contribution to the OS, and the number of points for each variable is determined by drawing a line upward to the points axis. The total score is obtained by summing up the scores of all variables on the corresponding scale, which can be used intuitively to estimate the probability of survival. Specifically, the projected total score on the bottom scales represents the likelihood of 1-, 3-, and 5-year survival. For instance, a patient with other organ metastasis, aged 56 years (≤58), LDH level of 340 U/L (≤357), GLB level of 29.9 g/L (>28.09), WBC level of 10 × 10^9^/L (≤10.3), MCV level of 80 fl (>78.55), MCH level of 28 pg (>25.24), and MO% level of 10.2% (≤11.42) has a total score of 116, indicating an estimated 1-, 3-, and 5-year overall survival of 81.5%, 56%, and 43%, respectively. Moreover, it could be found that MCH has the greatest impact on the OS prediction, followed by MCV from this nomogram.

### Evaluation of the OS predicting nomogram

**Figure 4.**
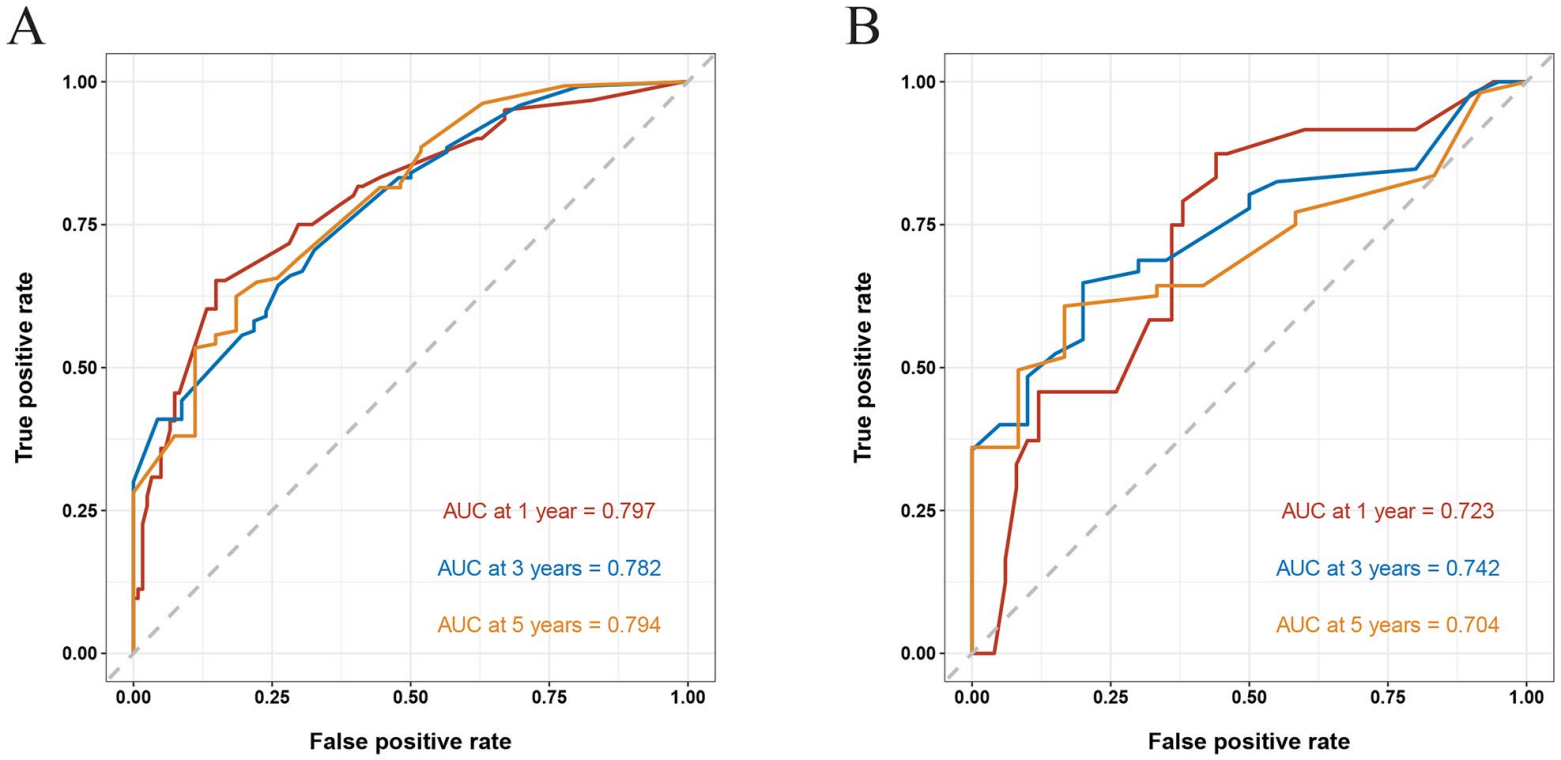
Receiver operating characteristic (ROC) curves of 1, 3, and 5-year in the training (A) and validation cohorts (B), respectively. The area under the ROC curve (AUC) was 0.797, 0.782, and 0.794 in the training cohort, and 0.723, 0.742, and 0.704 in the validation cohort, respectively.

As shown in Figure 4, the AUC of the nomogram predicting 1, 3, and 5-year OS was 0.797, 0.782, and 0.794 in the training cohort, and 0.723, 0.742, and 0.704 in the validation cohort, respectively. Meanwhile, the C-index of this model was 0.732. Obviously, both AUC and C-index over 0.7 suggested that our nomogram had moderate prediction efficacy.

**Figure 5.**
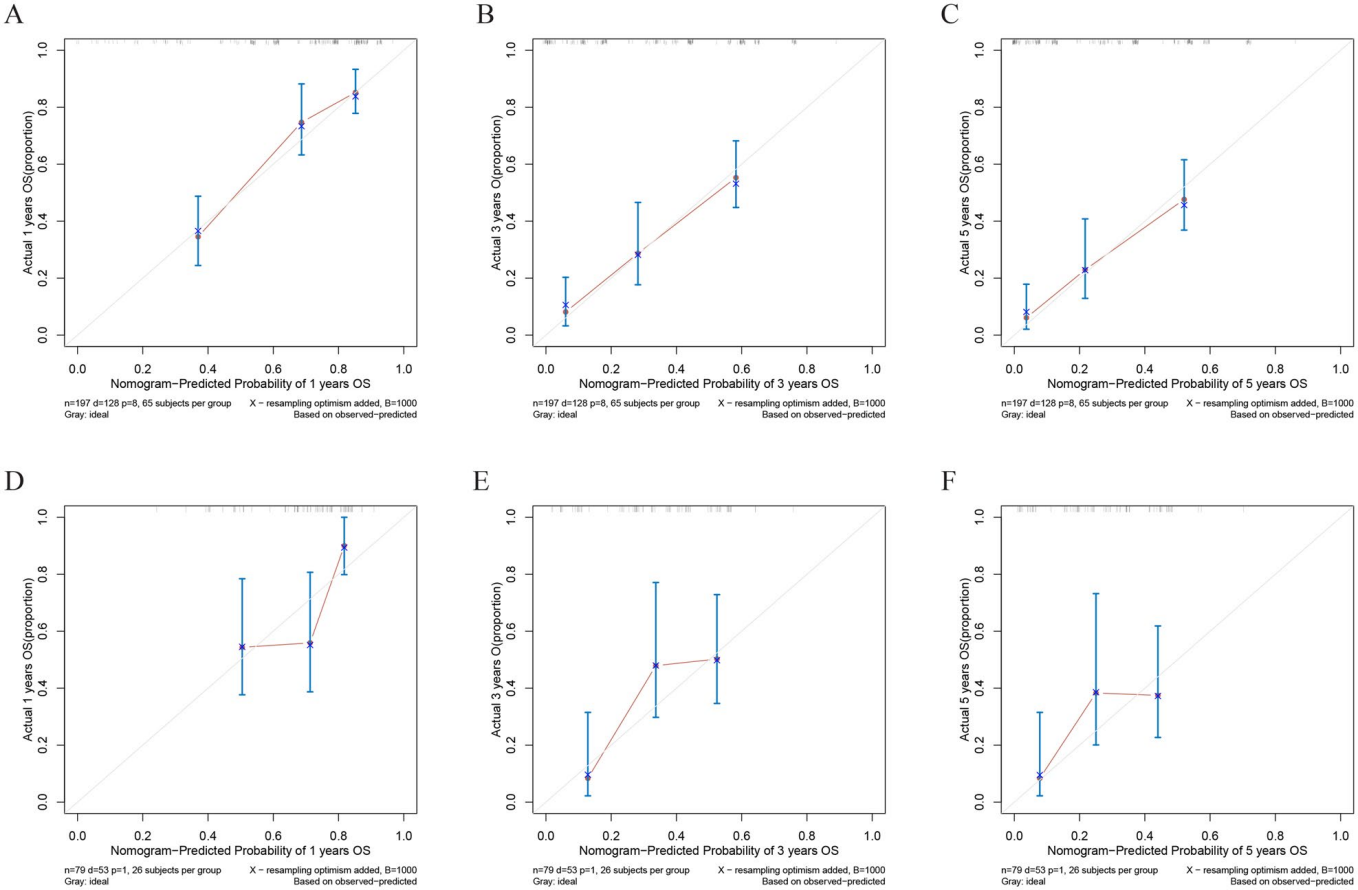
The calibration curves of the prognostic nomogram for the 1, 3, and 5-year OS prediction of the training (A–C) and validation group (D–F). the calibration curves suggested that the predictive outcome have good accordance with the actual 1, 3, and 5-year OS in both training and validation group.

**Figure 6.**
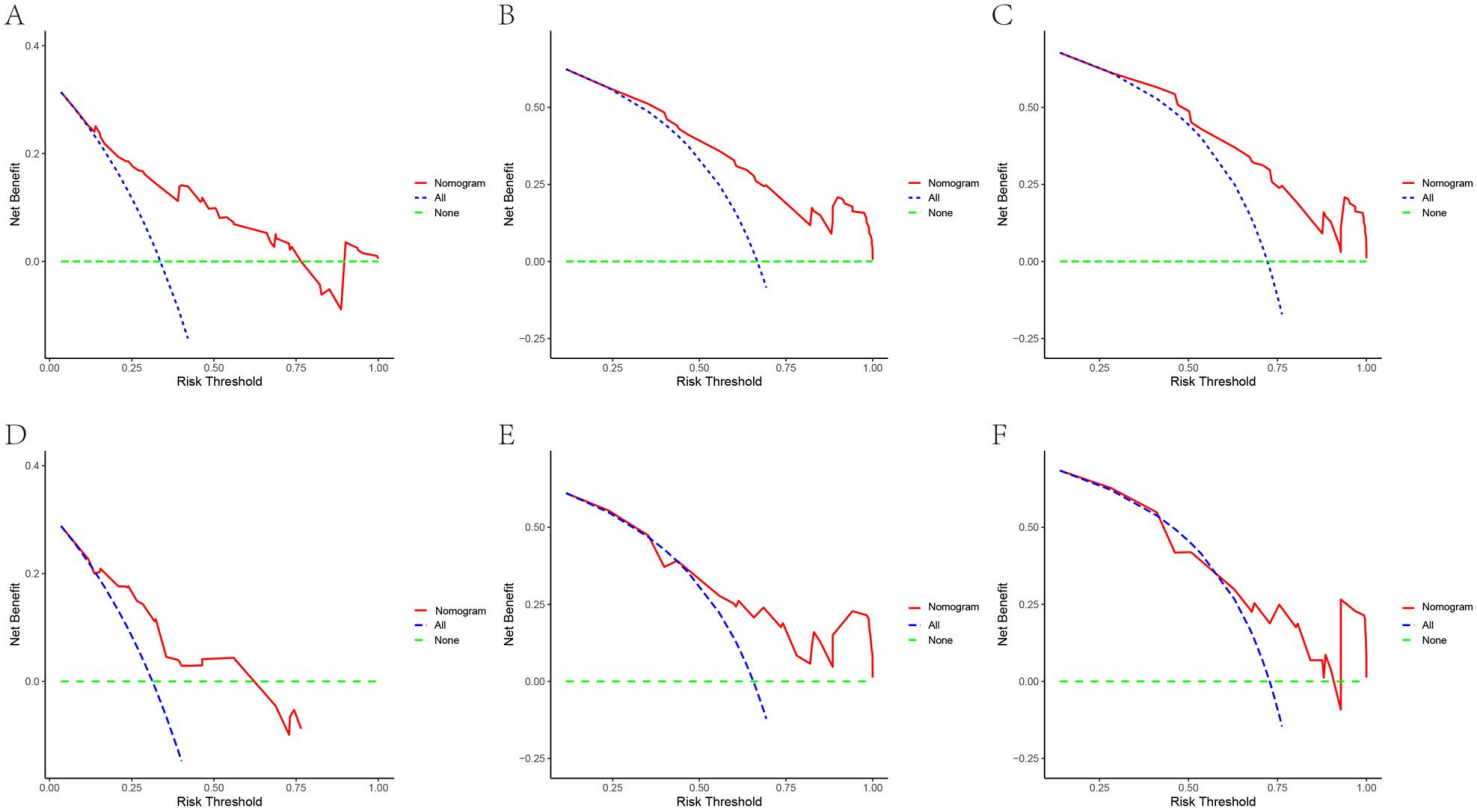
DCA of the nomogram for the survival prediction of breast cancer patients with bone metastasis in the training and validation groups. (A–C) 1, 3, and 5-year survival benefit in training cohort; (D-F) 1, 3, and 5-year survival benefit in validation cohort. The ‘ALL’, ‘none’ and ‘nomogram’ lines are represented as ‘intervention for all’ (blue dashed line), ‘intervention for none’ (green dashed line), and ‘result for the nomogram’ (red line). the ‘none’ and ‘ALL’ lines would show the expected net benefit without and with the intervention development respectively. DCA curves showed that nomogram manifested a higher net clinical benefit than ‘none’ and ‘all’.

In both training and validation cohorts, the calibration curves were generated to suggest that the nomogram-predicted OS had good accordance with the actual OS (Figure 5). Similarly, the DCA curves showed that our model had potential clinical utility (Figure 6). It was easily seen that our prediction model had the highest benefit across a wide range of preference values.

**Figure 7.**
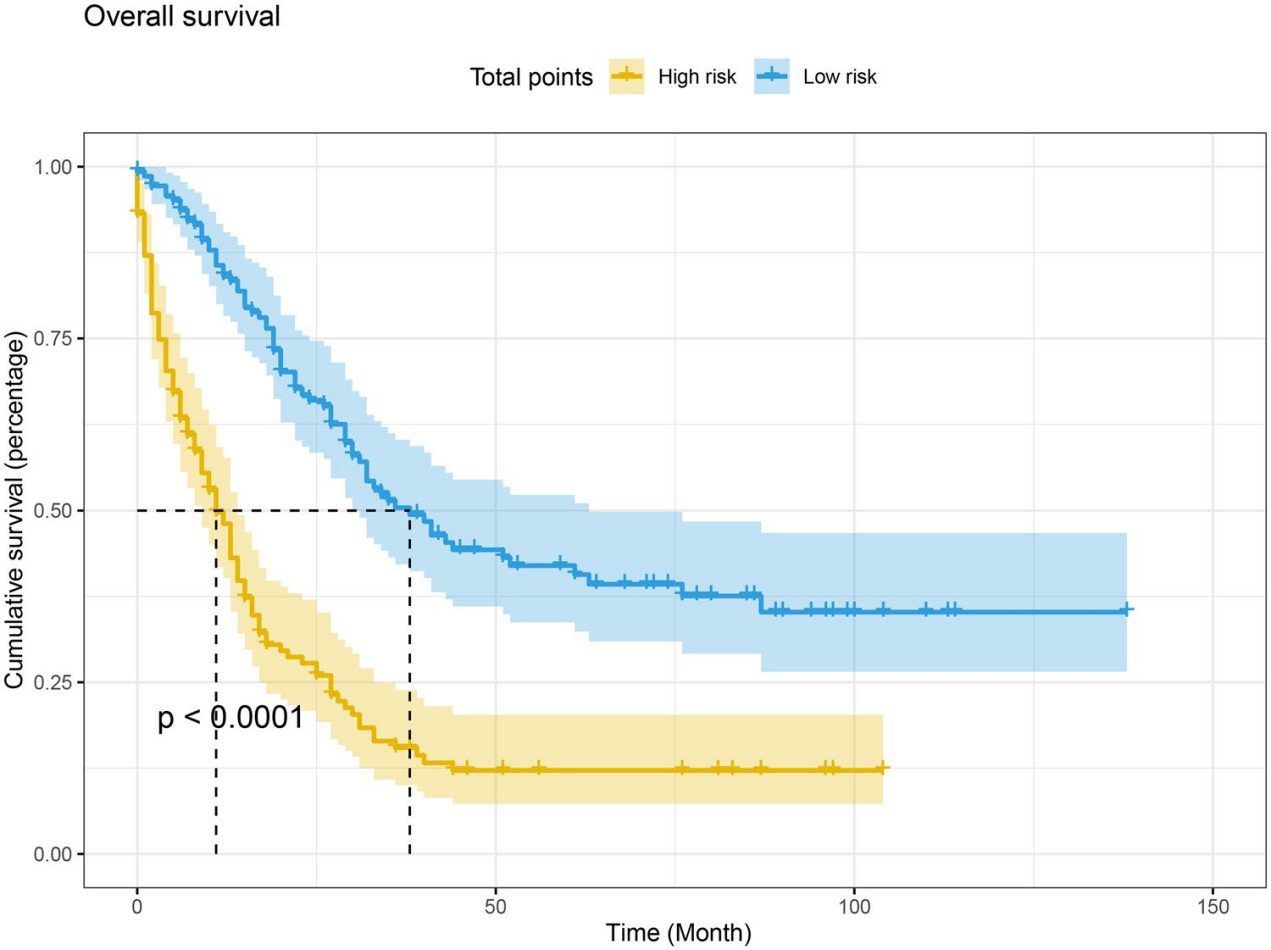
Kaplan-Meier curve of risk stratification for OS based on nomogram. The low-risk and high-risk meant normalized prognostic scores ≤ 1.93 and >1.93 for OS, respectively. Log-rank test was applied to estimate the significant difference.

After that, we obtained the normalized prognostic scores derived from the Cox model and grouped patients into low-risk and high-risk groups based on the median value of these scores. Specifically, patients with a normalized prognostic score less than or equal 1.93 were classified as low-risk, while those with a score greater than to 1.93 were classified as high-risk. Kaplan-Meier survival analysis and log-rank tests were then used to compare the overall survival (OS) between these two groups. Our findings revealed that patients in the high-risk group had a significantly shorter OS compared to those in the low-risk group (*p* < .0001, as shown in Figure 7).

## Discussion

Although great progress has been made in the diagnosis and treatment of BC, the occurrence of bone metastasis still brings great challenges to the clinicians. Advanced BC is regarded as incurable with current therapeutic strategy [22], and treatment goals are to improve the quality of life, control the complications, and prolong survival time by various treatment methods. For the clinicians, the more personalized treatments for each patient and more cost-effective healthcare resources may be driven by better understanding of prognosis. As we have known, the blood and biochemical tests are the routine examinations that almost all patients will undergo at admission. However, there were few studies focusing on the prognosis of BC bone metastasis through them. Herein, we developed and validated a prognosis predictive nomogram including clinical laboratory indicators to help clinicians to preliminarily assess the survival of patients in a convenient way. We found that eight independent risk factors were significantly related to prognosis, including age, other organ metastatic sites, LDH, GLB, WBC, MCV, MCH, and MO%.

Among our study, the prognosis of patients with additional organ metastatic sites was usually poorer than that of patients with bone-only metastasis. Consistently, studies have demonstrated that patients with bone-only metastasis have prolonged survival and a better prognosis than those with additional visceral metastases [23–26].

Previous analyses about prognosis of BC patients with bone metastasis clarified that HR+/Her2+ subtype was an independent protection factor of prognosis, and indicated the most favorable prognosis among all subtypes [7]. Subsequently, Lyu et al. [27] identified that PR positivity was also a protective prognosis factor in Her2+ BC patients with bone metastasis. In the total population of bone metastases from BC, our study found a significant and protective correlation between ER and prognosis in investigating the prognostic value of ER, PR, HER2 status for BC bone metastasis.

The development, progression and prognosis of malignancies were closely related to the systemic inflammation, immunity, and nutrition status of patients [28,29]. Anemia and malnutrition would impair the quality of life and prognosis of cancer patients [30]. Recently, Suzuki et al. [31] identified the significant effect between inflammation and nutritional indicators and prognosis in colorectal cancer. In the biochemical and blood routine examination, there are many indicators that not only reflect the patient’s internal environmental conditions but also the nutritional and inflammatory status. In our constructed OS predicted nomogram, we found that low MCH and elevated LDH, GLB, WBC, MCV, and MO% levels were poor prognostic factors.

MCH means the average amount of hemoglobin content in each red blood cell. With regarding to MCH as a prognostic factor, various studies have reached conflicting conclusions. Recently, Liang et al. [32] proved that high MCH was a risk factor in predicting the survival of esophageal squamous cell carcinoma and believed that this prognostic mechanism was related to the iron metabolism. Another study has shown that low HGB, MCH, and MCHC level were associated with unfavorable prognosis of resected lung cancer in univariate analysis [33], which is consistent with our results. The usage of different cut-off values, heterogeneous study populations, and varying sample sizes may be the reasons for these inconsistent results. Although our study recognized the high levels of HGB, MCH and MCHC have the positive influence with the prognosis of BC patients with bone metastasis in the univariate cox regression analysis, only MCH was the independent prognostic factor for BC bone metastasis rather than HGB and MCHC in the multiple analysis.

Significantly, the high level of MCV in the univariate Cox regression analysis was determined as a protective factor but as a risk factor in multiple Cox regression analysis. This phenomenon indicated that there were one or more potential confounding factors confusing the true effect of MCV in the univariate analysis, and revealing it as an independent prognostic risk factor after adjustment of multiple analysis. The result of MCV in our final model was consistent with a previous study that preoperative MCV was a poor prognostic factor for esophageal cancer [34].

Inflammation is a recognized hallmark feature of cancer, which plays an important role in the development and progression of malignant tumors, including BC [29,35]. It was reported that globulin can reflect immune and inflammatory status, and hyperglobulinemia may be an indicator of chronic inflammation in cancer patients [36,37]. LDH, a key enzyme involved in glycolysis, played a significant role in the process of metabolism of neoplastic cells, and its elevation showed the formation of tumor-derived immunosuppression [38]. In previous studies, high serum LDH has been identified containing the negative value on prognosis, including BC bone metastasis [8,39]. In our study, we also found that high level of globulin and LDH might indicate the poor OS, which were consistent with the above studies.

WBC are widely used peripheral blood indicators to reflect the systemic inflammation. A retrospective study with 6668 patients determined the prognostic value between low WBC and high LY% and the poor prognosis of non-metastatic nasopharyngeal carcinoma [13]. Similarly, our research has reached the consistent results in BC patients with bone metastasis. In the process of tumor immune response, changes will happen in the ratio of neutrophils, lymphocytes, and monocytes in peripheral blood [40,41]. There were many studies about the predictors related to immunity of the predictive efficacy in cancers, for example, lymphocyte to monocyte ratio (LMR), and these studies indicated low LMR level was communicated with poor prognosis of cancers [42,43]. The low LMR means monocyte dominates, and conversely, the lymphocyte dominates. It was consistent with our results which identified that MO% was an independent risk factors in the multivariate Cox regression.

In our study, almost all of the enrolled patients received therapy for primary BC before bone metastasis, such as radiotherapy, chemotherapy, endocrine therapy and surgery. Although different treatments for primary BC may influence the levels of blood indicators, we considered blood indicators tested at the time of bone metastasis diagnosis as baseline level to investigate the association with prognosis of BC bone metastasis, which was consistent with those previous studies [8,44]. On the other hand, our previous univariate Cox regression analysis showed that chemotherapy and surgery did not have a statistically significant impact on the OS of patients who received these treatments compared to those who did not receive them. To further evaluate the potential impact of treatment modalities on the OS, we excluded two patients who did not receive any therapy and conducted similar analyses in the training cohort as before. We found that the results (data not shown) were consistent with our present findings (Table 2), which revealed the therapy type of primary BC was not an independent risk factor for OS and our final model has relatively good stability.

There were several unavoidable limitations in our study. First, this was a single-center retrospective study of which model validation was based on internal validation *via* random grouping. Second, although the potential associations between the variables and prognosis were shown in the final model, the relevant mechanism could not be absolutely explained and needed further investigation. Third, given the retrospective nature of the study, there was inevitable incomplete data in this study, such as operation patterns and the detail of chemotherapy protocol. Fourth, the cases requirement for prognostic prediction model was general more than 15–20 times the number of independent variables in multivariate Cox analysis [45]. In our study, 20 variables analyzed in multivariate Cox analysis need at least 300 enrolled cases. This means our sample size of 276 enrolled patients was not enough, which would diminish the statistical effectiveness. Therefore, larger sample validation studies should be conducted at different institutions to further validate the results of our study. These studies could determine the predictive ability of blood routine and biochemical tests for the prognosis of bone metastasis in BC patients and offer more comprehensive and reliable data to confirm the prognostic value and clinical significance of these laboratory tests. Fifth, although the nomogram demonstrated prognostic value for BC bone metastasis in both the training and validation cohorts, but the predictive capabilities of the validation cohort appeared to be inferior to that of the training cohort. This result may be attributed to the smaller sample size of patients or different sample sources in the validation cohort, which can introduce bias. In addition, there were differences noted in the proportions of patients with different clinical features between the two study groups, which might also have influenced the discriminative ability of the nomogram. In fact, studies on prognostic model construction often report varying results across different cohorts due to differences in sample sizes and patient demographics [46,47]. Therefore, the verification of our prognostic nomogram is necessary in larger, diverse cohorts in future study. Finally, our study involved patients who had undergone routine examination before the treatment for bone metastasis. These prognostic factors may not be fully suitable for patients with treated bone metastases.

In conclusion, in this study, we developed a prognostic nomogram of predicting OS of BC patients with bone metastasis, and our study suggested that two clinical features (age and other organ metastasis sites) and six clinical laboratory examination indicators (LDH, GLB, WBC, MCV, MCH, and MO%) could estimate the prognosis among the patients who were undergoing BC bone metastasis. Our model employed a blend of clinical features and laboratory examination indicators that reflect various aspects of a patient’s condition, resulting in enhanced prediction accuracy. Moreover, the Cox regression model results were expressed through a nomogram visualization tool, offering simple and direct interpretation for the clinical doctors. Our nomogram model might assist doctors in assessing the risk factors and prognosis of BC patients with bone metastasis. It might be suggested that patients at high risk based on the nomogram may require more aggressive treatment strategies.

## Data Availability

The raw data and the R codes supporting the conclusions of this article will be made available by the corresponding authors, without undue reservation.
